# *Salix gordejevii* females exhibit more resistance against wind erosion than males under aeolian environment

**DOI:** 10.3389/fpls.2022.1053741

**Published:** 2022-11-14

**Authors:** Shaowei Ma, Guohou Liu, Lei Wang, Guanzhi Liu, Xiao Xu

**Affiliations:** ^1^ College of Life Science and Technology, Inner Mongolia Normal University, Hohhot, China; ^2^ College of Grassland, Resources and Environment, Inner Mongolia Agricultural University, Hohhot, China; ^3^ Key Laboratory of Southwest China Wildlife Resources Conservation (China West Normal University), Ministry of Education, Nanchong, China

**Keywords:** wind erosion, dioecy, sexual difference, plant growth, physiological characteristic, *Salix gordejevii*

## Abstract

Effects of wind erosion on growth and adaptability have been widely reported in many plants, but little attention has been paid to dioecious plants. Recent studies have shown that sex-specific responses to environmental changes in many plants exist. To explore sexual differences in response to wind erosion, female and male *Salix gordejevii* saplings growing on inter-dune land (no erosion) and on the windward slope of the dune (20cm wind erosion) in Hunshandake Sandy Land were chosen and their morphology, biomass and physiological traits were investigated, respectively. Wind erosion significantly reduced plant growth, biomass accumulation, gas exchange and chlorophyll fluorescence, and obviously disrupted osmotic regulation function and antioxidant enzyme system in both sexes, especially in males. Under wind erosion condition, females exhibited higher sapling height (SH), basal diameter (BD), leaf dry mass (LDM), root dry mass (RDM), total dry mass (TDM), root percentage in total dry mass, net photosynthesis rate (*P*_n_), maximum efficiency of photosystem II (*F*_v_/*F*_m_), effective quantum yield of PSII (*Φ*_PSII_), relative water content (RWC) of leaves, superoxide dismutase (SOD) and peroxidase (POD) activities, but lower malondialdehyde (MDA), proline as well as soluble sugar content than did males. However, no significant sexual differences in most of these traits were observed under no erosion condition. Our results demonstrated that females possess a greater resistance to wind erosion than do males, with females having a better photosynthetic capacity, stronger water retention capacity and more efficient antioxidant system to alleviate negative effects caused by aeolian environment.

## Introduction

Aeolian environment is regarded as one of the extreme environments that seriously affect plant growth and reproduction in the dune ecosystem of arid and semi-arid areas (Shi et al., 2004; Dech and Maun, 2006; Perumal and Maun, 2006; Gilbert and Ripley, 2010; Tang et al., 2016; Fan et al., 2018). Affected by windblown sand movement, plants growing on the leeward slopes of dunes often suffer partial or complete sand burial, whereas those on the windward slopes of dunes experience varying degrees of wind erosion (Luo and Zhao, 2015; Luo et al., 2018). During this process, wind erosion directly affects plant survival and growth by physically damaging root system (e.g. root distortion, splitting and breaking) and exposing them to the air, thereby greatly impairing efficiency of water and nutrient uptake and also photosynthesis (Yu et al., 2008; Liu et al., 2014a; Liu et al., 2014b). Previous studies have reported that morphological growth and biomass accumulation of plants are significantly inhibited under wind erosion environment (Yu et al., 2008; Li et al., 2010; Liu et al., 2014a). Photosynthesis, including net photosynthetic rate and chlorophyll fluorescence parameters, also shows similar trends (Fan et al., 2018). Adjustments in morphological and physiological characteristics constitute the important means by which plants increase their resistance and adaptability (Anten et al., 2005; Liao et al., 2020). However, little attention has been paid to the physiological responses of plants to changes in the surrounding environment caused by wind erosion.

Dioecious plants have been considered to be a consequence of different requirements for disseminating pollen and producing fruits and seeds, and play key roles in maintaining the function of terrestrial ecosystems (Renner and Ricklefs, 1995; Obeso, 2002). In recent years, the survive pressure of plants is being expanded by global warming, climate change, and environmental pollution. Sexual differences in morphology, physiology and biochemistry of dioecious plants have been documented in previous studies under various environmental stresses, including drought, extreme temperatures, UV radiation, nutrient deficiency or heavy metals (Juvany and Munne-Bosch, 2015; Melnikova et al., 2017). In some species, such as *Populus cathayana*, *P. yunnanensis* and *Morus alba*, males are better adapted to the majority of the stress conditions than females, exhibiting less damage, better growth, higher photosynthetic capacity and antioxidant activity (Xu et al., 2008; Zhang et al., 2012; Jiang et al., 2013; Chen et al., 2016). In contrast, the opposite has been observed in other species, such as *Acer negundo, Salix myrsinifolia, S. paraplesia* (Ward et al., 2002; Randriamanana et al., 2015; Jiang et al., 2016). These studies indicated that female and male plants’ growth and physiological response to environmental stress are different, while the performance of females and males in stressful environments may be related to the species.

*Salix* species are common dioecy and have characters of fast-growing, adaptable and easy to cultivate, which are pioneer tree species used in ecological stability and vegetation restoration in the terrestrial ecosystem, especially in the dune ecosystem of arid and semi-arid areas (Liu et al., 2003; Yan et al., 2007; Kubo et al., 2013). Despite sex-specific responses to environmental stress (e.g., long-term UV-B radiation, nutrient-poor, water deficiency and nocturnal warming) in *Salix* were found to exist in some studies (Randriamanana et al., 2015; Jiang et al., 2016; Liao et al., 2019; Liao et al., 2020), no sex-specific data are available with regard to the morphological or physiological characteristics responses to wind erosion. In addition, *Salix* and *populus* are sister genera in the Salicaceae family, but *Salix* females and *Populus* males outperform the opposite sex under environmental stress. And the reason for this inconsistency remains obscure. Therefore, *Salix gordejevii* Y. L. Chang et Skv., a dioecious plant widely distributed in Hunshandake Sandy Land of China, was chosen and sexual differences in morphological growth, biomass accumulation, photosynthetic capacity, related biochemical material content and antioxidant enzyme activity between male and female saplings were investigated. The goals of this study were to: (a) determine the effects of wind erosion on morphological and physiological characteristics in *S. gordejevii*; (b) evaluate whether females exhibit more resistance against wind erosion than males; (c) analyze the possible reasons for inconsistent sexual performance related to species in Salicaceae.

## Materials and methods

### Study area

The study was conducted in the hinterland of Hunshandake Sandy Land (41°56′ − 43°11′ N, 115°00′ − 116°42′ E), which is located in Zhenglan banner of Xilin Gol league of Inner Mongolia Autonomous Region, China. The climate type is a mid-temperate continental monsoon climate, with a long cold winter and a short warm summer. The mean annual temperature, precipitation and evaporation are 1.7°C, 355 mm and 1931.4 mm, respectively. The mean annual evaporation is much larger than the mean annual precipitation. The frost-free season lasts approximately 110 days. The annual mean wind speed is about 4 m·s^-1^, with the main wind direction being northwest wind and frequent sandstorms in spring and autumn. The soil type in the area is chestnut soil, which is distributed with aeolian sandy soil. Dominant plant species include xylophyta such as *Ulmus pumila* var. *sabulosa*, *S. gordejevii*, *S. microstachya* var. *bordensis*, *Artemisia halodendron*, and perennial herbage such as *Potentilla acaulis*, *Leymus chinensis*, *Cleistogenes squarrosa*, etc. (Su et al., 2009).

### Experimental design

The experiment was carried out in an area of semi-fixed dune of Hunshandake Sandy Land, with vegetation dominated by *S. gordejevii* saplings that were artificially planted by the local forestry bureau in 2015. When planting, the cuttings (about 50 cm long) were inserted 45 cm into the soil with a 5-cm height above the soil surface. In April 2018 (the flowering season of *S. gordejevii*), we identified and labeled the sex of saplings growing on inter-dune land and on the windward slope of the dune, respectively. In August, we selected experimental materials for control group and wind erosion group in each habitat, according to the exposure of cuttings that were used for planting in 2015. Among them, the exposed degree of cuttings in control group was still 5-cm height above the soil surface (no erosion), while that in wind erosion group was about 25-cm height (20 cm wind erosion). 20 male and 20 female saplings in each condition were selected and used for growth and physiological index analysis. The experimental layout was completely randomized with two factors (sex and wind erosion). There were finally four conditions: (1) females with no erosion (control); (2) males with no erosion (control); (3) females with 20 cm erosion (wind erosion); and (4) males with 20 cm erosion (wind erosion).

### Morphological and biomass traits measurements

Sapling height (SH), basal diameter (BD) and crown size (CS) of each sapling were measured on 22 August, 2018. Then all saplings were harvested and divided into leaves, stems and roots after washing out of soil. After oven-dry to constant weight at 70°C for 48 h, leaf dry mass (LDM), stem dry mass (SDM) and root dry mass (RDM) were weighed and recorded. The total dry mass (TDM) was calculated as the sum of leaf, stem and root dry mass. The percentage in total dry mass was calculated for leaf, stem and root, respectively. All biomass did not include the weight of cuttings that were used for planting in 2015.

### Gas exchange and chlorophyll fluorescence measurements

Three male and female plants of each condition were randomly selected, and the fourth fully expanded leaf from apex of each plant was selected for gas exchange and chlorophyll fluorescence measurements on August 21. Net photosynthetic rate (*P*_n_), stomatal conductance (*G*_s_), intercellular CO_2_ concentration (*C_i_*) and transpiration rate (*T_r_*) were measured between 09:00 a.m. and 11:30 a.m. with a LI-6400 portable photosynthesis system (LI-COR, Lincoln, NE, USA) under the following conditions: leaf temperature, 26°C; photosynthetic photon flux (PPF), 1500 µmol m^-2^ s^-1^; relative air humidity, 30%; and ambient CO_2_ concentration, 380 ± 10 µmol mol ^-1^. Data were recorded once steady-state gas exchange rates were observed.

Chlorophyll fluorescence kinetics parameters (*F*_v_/*F*_m_, maximum efficiency of photosystem II (PSII) and *Φ*_PSII,_ effective quantum yield of PSII) were measured with a LI-6400 chlorophyll fluorescence system (LI-COR, Lincoln, NE, USA). The leaf samples were placed in dark treatment for 30 min using aluminum foils cover, and the minimum fluorescence (*F*_0_) and the maximum fluorescence (*F*_m_) were measured by low modulated light and a saturating pulse, respectively. The *F*_v_/*F*_m_ was then calculated using the equation: *F*_v_/*F*_m_ = (*F*_m_ − *F*_0_)/*F*_m_ (Brugnoli and Bjorkman, 1992). Subsequently, the leaves were exposed to a PPFD of 1000 µmol m^-2^ s^-1^ for 15 min to measure the maximum light-adapted fluorescence (*F*_m_′) and the steady-state fluorescence (*F*_s_). The *Φ*_PSII_ was then calculated using the equation: *Φ*_PSII_ = (*F*_m_′ − *F*_s_)/*F*_m_′ (Brugnoli and Bjorkman, 1992).

### Relative water content and malondialdehyde content determination

Three fully expanded and exposed leaves were randomly selected from each condition for relative water content (RWC) and malondialdehyde (MDA) content determination. RWC was determined by the method of Weatherley (1950). The leaves were cut into about 1 cm segments and weighed fresh weight (*W_f_*), then were immersed in distilled water to saturation and weighed saturated weight (*W_s_*). Finally, the saturated leaves were dried to a constant weight (*W_d_*) at 70°C for 48 h. The formula for calculating RWC of leaves was the following:


$$\text{RWC}\left(\%\right)=100\times (W_{f}-W_{d})/(W_{s}-W_{d})$$

Malondialdehyde (MDA) content was determined by the method of Hodges et al. (1999). 0.5 g leaves of each sample were homogenized in 5 mL of 5% trichloroacetic acid (TCA) and centrifuged at 4 000g for 10 min. 2 mL supernatant was collected and added to 2 mL of 0.67% thiobarbituric acid (TBA). The mixture was heated in boiling water for 30 min followed by rapid cooling in an ice bath, and then centrifuged at 4 000 g for 10 min. The absorbance values of supernatant were measured respectively at 450 nm, 532 nm and 600 nm and converted to MDA content.

### Proline and soluble sugar content determination

Proline assay was measured according to the method of Bates et al. (1973). 0.5 g leaves were homogenized in 5 mL of 3% sulfosalicylic acid solution. After extracted in boiling water for 10 min and cooled, 2 mL of the extract was mixed with 2 mL of glacial acetic acid and 2 mL of acid ninhydrin solution in a test tube at 100°C for 30 min. After cooling, 4 mL of toluene was added to the mixture to extract the chromophore containing toluene, and the absorbance was measured at 520 nm.

Soluble sugar content was measured as described by Ebell (1969). 0.1g leaves of each sample were put into tube with 5 mL distilled water, which were extracted with sealing in boiling water for 30 min. The reaction mixture with a total volume of 7.5 mL contained 0.5 mL the extract, 1.5 mL distilled water, 0.5 mL enthrone ethyl acetate and 5 mL concentrated sulfuric acid. After the tube was placed in boiling water for 1 min and cooled, the absorbance value was measured at 630 nm.

### Superoxide dismutase and peroxidase activity determination

0.5 g fresh leaves of each sample were ground with a pre-cooled mortar and pestle with 50 mM potassium phosphate buffer (pH 7.8) to extract enzyme for the measurements of superoxide dismutase (SOD) and peroxidase (POD) activity (Maehly and Chance, 1954; Beauchamp and Fridovich, 1971). The samples were centrifuged at 12 000g at 4°C for 20 min, and the supernatants were used for enzyme activity assays. All operations were performed at 0 − 4°C.

The SOD activity was determined by measuring the ability to inhibit the photochemical reduction of nitroblue tetrazolium (NBT), as described by Beauchamp and Fridovich (1971). 0.05 mL supernatant was collected with 1.5 mL of 50 mM potassium phosphate buffer (pH 7.8) and distilled water 0.25 mL, and added 0.3 mL each of 130 mM methionine (Met), 750 µM NBT, 100 μM EDTA-Na_2_ and 20 μM riboflavin. After mixing enough, the mixture was carried out for 30 min under irradiance of 170 µmol photons m^-2^ s^-1^ provided by a white fluorescent lamp. The absorbance was measured at 560 nm using a non-illuminated identical tube as a blank. The SOD activity was expressed as unit g^-1^ FW.

The POD activity was determined by the method of Maehly and Chance (1954) with guaiacol as an electron donor, and the absorbance of the supernatant was measured at 470 nm for 3 min. The activity of enzyme was represented by the change of optical density per min. One enzyme unit was defined as the amount of enzyme causing an absorbance value change of 0.01 per min under standard conditions. The POD activity was expressed as unit g^-1^ FW min^-1^.

### Statistical analysis

SPSS 20.0 statistical software (SPSS, Chicago, IL, USA) was used for statistical analyses. The data were checked for normality and homogeneity of variances, and log-transformed to correct deviations from these assumptions when needed. Two-way ANOVA was used to evaluate the effects of sex, wind erosion and their interaction. Individual differences among means of different conditions were determined by Duncan’s multiple range test. Differences were considered significant at *P* < 0.05.

## Results

### Sexual differences in morphological growth and development

**Figure 1:**
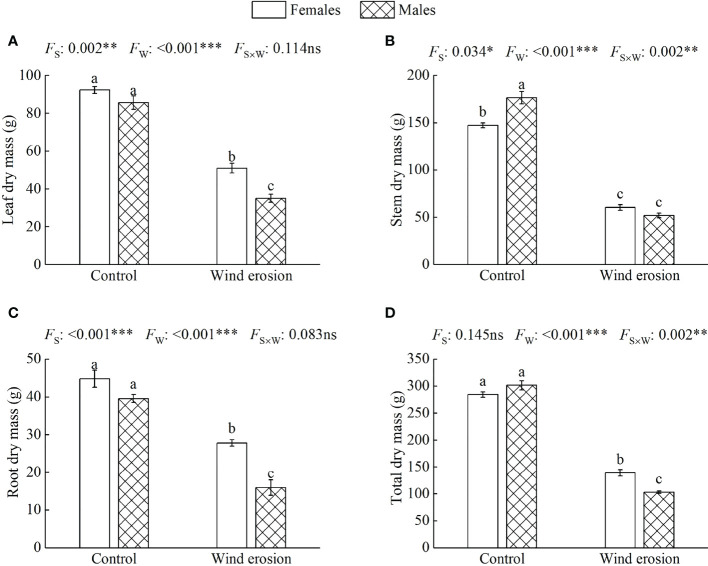
Leaf dry mass **(A)**, stem dry mass **(B)**, root dry mass **(C)** and total dry mass **(D)** in females and males of *S. gordejevii* as affected by wind erosion. Values are means ± SE (*n* = 3). Different letters above bars indicate statistically significant differences between conditions according to Duncan’s multiple rang test (*P* < 0.05). Significance values of the factorial analysis (ANOVA) for: *F*_s_, sex effect; *F*_w_, wind erosion effect; and *F*_s×w_, the interactive effect of sex and wind erosion are denoted as: ns, non-significant; ^*^ *P* < 0.05; ^**^ *P* < 0.01; ^***^ *P* < 0.001.

As shown in **Table 1**, wind erosion significantly decreased SH in all saplings (*P* < 0.001) but CS only in males compared with control. There were significant differences in SH and BD between sexes in the control condition, with females having higher SH and lower BD than males. Under wind erosion condition, females had significantly higher SH and BD than males, but there was no significant difference in CS between male and female saplings. Similarly, wind erosion significantly inhibited biomass accumulation of all saplings (*P* < 0.001), and induced females to exhibit higher LDM, RDM and TDM than males. While there were no significant differences in these traits (except for SDM) between the sexes in the control condition (**Figure 1**).

**Table 1:** Sapling height (SH), basal diameter (BD) and crown size (CS) in females and males of *S. gordejevii* as affected by wind erosion.

Condition	Sex	SH(cm)	BD (mm)	CS (m^2^)
Control	Females	160.33 ± 3.76a	7.34 ± 0.09b	0.64 ± 0.05ab
	Males	145.00 ± 2.89b	8.58 ± 0.42a	0.75 ± 0.09a
Wind erosion	Females	106.00 ± 3.79c	8.57 ± 0.13a	0.54 ± 0.05ab
	Males	82.00 ± 5.51d	7.12 ± 0.16b	0.50 ± 0.07b
	*P* > *F*_s_	<0.001***	0.674ns	0.611ns
	*P* > *F*_w_	<0.001***	0.654ns	0.032*
	*P* > *F*_s×w_	0.321ns	<0.001***	0.270ns

Values are means ± SE (*n* = 3). Different letters in the same column indicate statistically significant differences between conditions according to Duncan’s multiple rang test (*P* < 0.05). Significance values of the factorial analysis (ANOVA) for: *F*_s_, sex effect; *F*_w_, wind erosion effect; and *F*_s×w_, the interactive effect of sex and wind erosion are denoted as: ns, non-significant; ^*^ *P* < 0.05; ^***^ *P* < 0.001.

**Figure 2:**
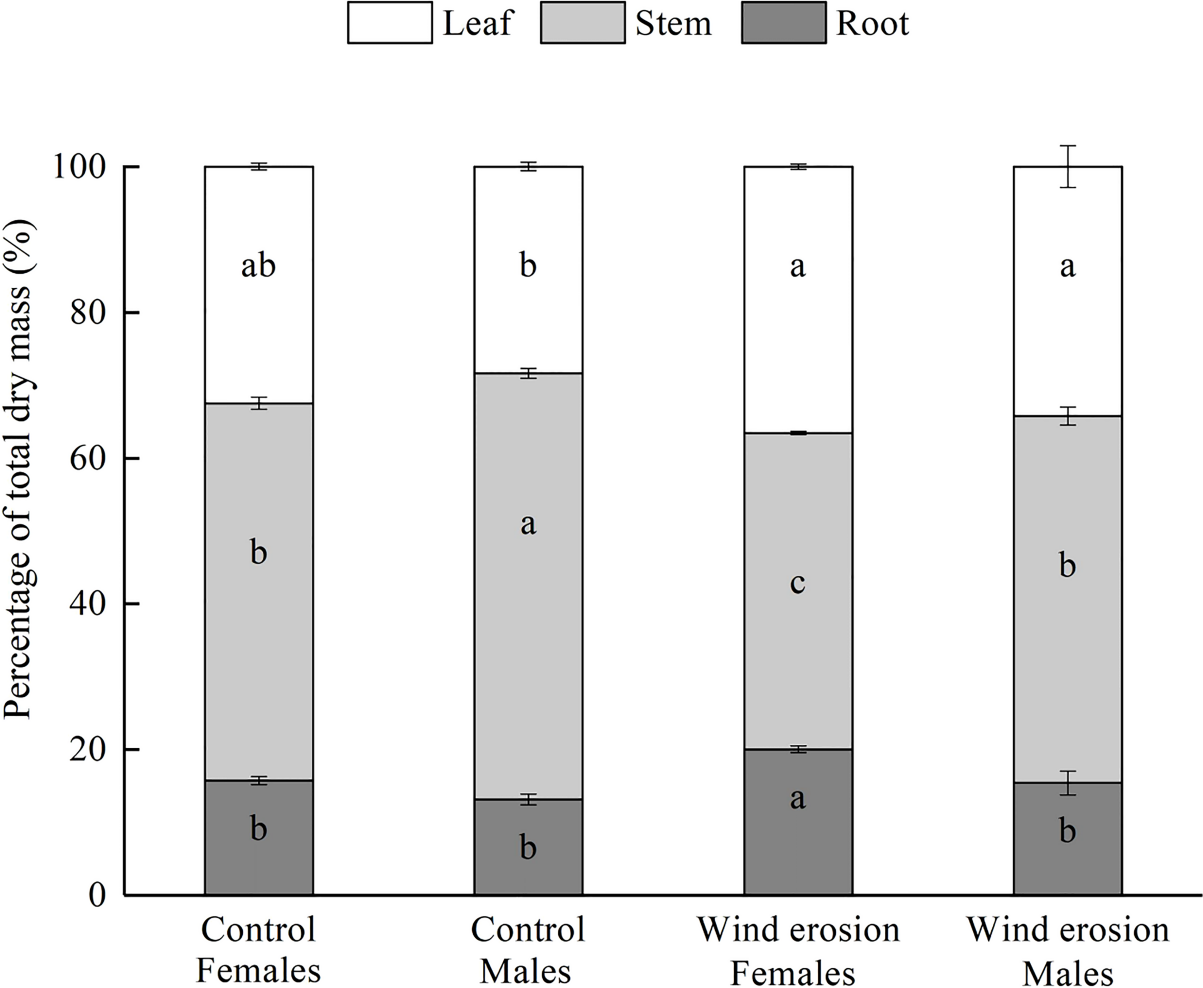
Biomass allocation in females and males of *S. gordejevii* as affected by wind erosion (means ± SE, *n* = 3). Different letters on bars of each color indicate statistically significant differences between conditions according to Duncan’s multiple rang test (*P* < 0.05).

Moreover, biomass allocation was also altered by wind erosion leading to a strong decrease in stem resource allocation of two sexes, and increase in root resource allocation of females and leaf resource allocation of males (**Figure 2**). Under the control condition, there were no significant differences in leaf and root percentage in total dry mass between two sexes. However, females had higher root percentage in total dry mass than males under wind erosion condition. Stem percentage in total dry mass were significantly always lower in females than in males under control and wind erosion condition.

### Sexual differences in gas exchange and chlorophyll fluorescence

Photosynthesis of all saplings was reduced markedly, and males and females differed in their responses to wind erosion, according to the changes of gas exchange and chlorophyll fluorescence parameters (**Table 2**). Compared with control, wind erosion significantly decreased *P*_n_, *C*_i_ and *T*_r_ in all saplings (*P* < 0.001). Except females always had significantly higher *P*_n_ than males, there were no significant sexual differences in *G*_s_, *C*_i_ and *T*_r_ both under control and wind erosion condition (**Table 2**). In addition, wind erosion had significant negative effects on *F*_v_/*F*_m_ only in males. There were no significant differences in *F*_v_/*F*_m_ and *Φ*_PSII_ between the sexes in the control condition. However, females had significantly higher *F*_v_/*F*_m_ and *Φ*_PSII_ than males under wind erosion condition (**Table 2**).

**Table 2:** Net photosynthesis rate (*P*_n_), stomatal conductance (*G*_s_), intercellular CO_2_ concentration (*C*_i_), transpiration (*T*_r_), maximum efficiency of photosystem II (*F*_v_/*F*_m_) and effective quantum yield of PSII (*Φ*_PSII_) in females and males of *S. gordejevii* as affected by wind erosion.

Condition	Sex	*P*_n_ (μmol m^-2^ s^-1^)	*G*_s_ (mol m^-2^ s^-1^)	*C*_i_ (μmol mol^-1^)	*T*_r_ (mmol m^-2^ s^-1^)	*F*_v_/*F*_m_	*Φ*_PSII_
Control	Females	14.58 ± 0.69a	0.15 ± 0.01a	295.89 ± 17.78a	5.31 ± 0.54a	0.76 ± 0.01a	0.28 ± 0.04ab
	Males	12.72 ± 0.50b	0.14 ± 0.03a	319.73 ± 10.09a	4.94 ± 0.81a	0.74 ± 0.01a	0.21 ± 0.01bc
Wind erosion	Females	8.42 ± 0.72c	0.15 ± 0.02a	206.37 ± 36.89b	2.68 ± 0.16b	0.75 ± 0.01a	0.32 ± 0.02a
	Males	5.28 ± 0.17d	0.12 ± 0.02a	186.55 ± 20.08b	1.98 ± 0.34b	0.63 ± 0.01b	0.18 ± 0.01c
	*P* > *F*_s_	0.002**	0.480ns	0.934ns	0.336ns	<0.001***	0.002**
	*P* > *F*_w_	<0.001***	0.668ns	<0.001***	<0.001***	<0.001***	0.945ns
	*P* > *F*_s×w_	0.288ns	0.668ns	0.377ns	0.754ns	<0.001***	0.175ns

Values are means ± SE (*n* = 3). Different letters in the same column indicate statistically significant differences between conditions according to Duncan’s multiple rang test (*P* < 0.05). Significance values of the factorial analysis (ANOVA) for: *F*_s_, sex effect; *F*_w_, wind erosion effect; and *F*_s×w_, the interactive effect of sex and wind erosion are denoted as: ns, non-significant; ^**^P < 0.01; ^***^P < 0.001.

### Sexual differences in physiological characteristics

**Figure 3:**
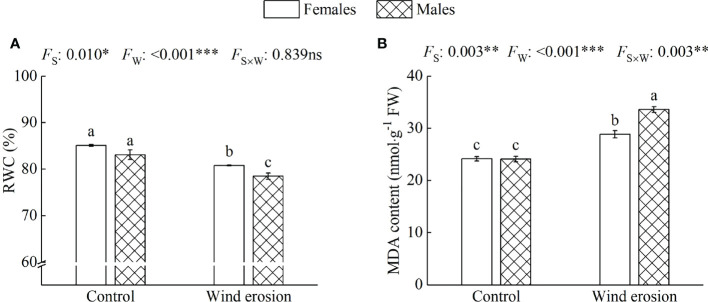
Relative water content (RWC) of leaves **(A)** and malondialdehyde (MDA) content **(B)** in females and males of *S. gordejevii* as affected by wind erosion. Values are means ± SE (*n* = 3). Different letters above bars indicate statistically significant differences between conditions according to Duncan’s multiple rang test (*P* < 0.05). Significance values of the factorial analysis (ANOVA) for: *F*_s_, sex effect; *F*_w_, wind erosion effect; and *F*_s×w_, the interactive effect of sex and wind erosion are denoted as: ns, non-significant; ^*^P < 0.05; ^**^P < 0.01; ^***^P < 0.001.

**Figure 4:**
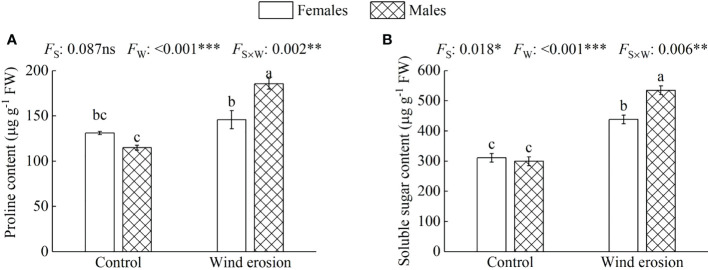
Proline content **(A)** and soluble sugar content **(B)** in females and males of *S. gordejevii* as affected by wind erosion. Values are means ± SE (*n* = 3). Different letters above bars indicate statistically significant differences between conditions according to Duncan’s multiple rang test (*P* < 0.05). Significance values of the factorial analysis (ANOVA) for: *F*_s_, sex effect; *F*_w_, wind erosion effect; and *F*_s×w_, the interactive effect of sex and wind erosion are denoted as: ns, non-significant; ^*^P < 0.05; ^**^P < 0.01; ^***^P < 0.001.

**Figure 5:**
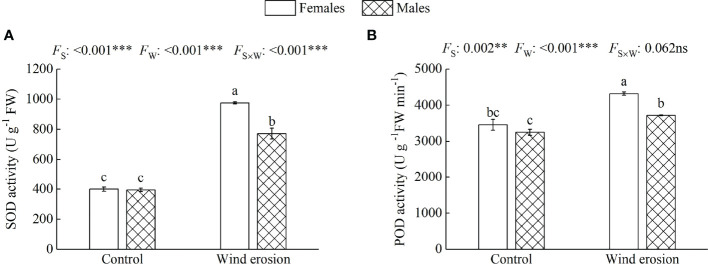
Superoxide dismutase activity (SOD) **(A)** and peroxidase activity (POD) **(B)** in females and males of *S. gordejevii* as affected by wind erosion. Values are means ± SE (*n* = 3). Different letters above bars indicate statistically significant differences between conditions according to Duncan’s multiple rang test (*P* < 0.05). Significance values of the factorial analysis (ANOVA) for: *F*_s_, sex effect; *F*_w_, wind erosion effect; and *F*_s×w_, the interactive effect of sex and wind erosion are denoted as: ns, non-significant; ^**^P < 0.01; ^***^P < 0.001.

Similar to plant growth and photosynthesis, physiological characteristics of both sexes, including RWC, MDA content, osmotic regulation function and antioxidant enzyme system, were obviously disrupted by wind erosion (**Figures 3**–**5**). Wind erosion significantly decreased RWC of leaves and increased MDA content in all saplings (*P* < 0.001). When comparing the sexes, females exhibited significantly higher RWC but lower MDA content than did males under wind erosion conditions, whereas there were no significant sexual differences under the control conditions (**Figure 3**). Furthermore, wind erosion significantly induced the increase of soluble sugar content, SOD and POD activity in all saplings (*P* < 0.001) but proline content only in males. Females had lower the content of proline and soluble sugar, but higher the activities of SOD and POD than males under wind erosion conditions. However, no significant sexual differences were detected in these traits under the control conditions (**Figures 4**, **5**).

## Discussion

### Wind erosion inhibited growth and photosynthetic capacity more in males than in females

Wind erosion, one of the extreme environments of dune ecosystem, causing serious negative effects on plant morphology, growth and development have been reported in many plants, such as root exposure, water loss, leaf area reduction, photosynthetic capacity decline, etc. These adverse effects ultimately lead to severe limitation of biomass accumulation and even death (Levin et al., 2008; Liu et al., 2014a; Liu et al., 2014b; Luo and Zhao, 2015). Similar results were also confirmed by our study showing that SH, CS and dry biomass of leaf, stem and root reduced significantly in the wind erosion condition (**Table 1**, **Figure 1**), which indicated that morphological development and biomass accumulation of *S. gordejevii* were severely hindered by wind erosion. Meanwhile, we found that males and females differed in their responses to wind erosion. Compared with the control condition, the SH, CS and SDM of females decreased 33.89%, 14.47% and 58.90% respectively, which were significantly lower than that of males about 43.45%, 33.28% and 70.62% (**Table 1**, **Figure 1**). Females presented significantly higher LDM, RDM and TDM than males during wind erosion, whereas no significant sexual differences in these traits were observed in the control condition (**Figure 1**). These results demonstrated that wind erosion inhibited morphological growth and biomass accumulation more in males than in females, which is in accordance with previous findings in *Salix* species under environmental stress (Jiang et al., 2016; Liao et al., 2019; Liao et al., 2020). In addition, we observed that wind erosion significantly increased BD in females but decreased BD in males compared with the control condition (**Table 1**). We think this is due to females have higher CS and SDM than males under wind erosion condition, which cause them to develop stronger stem and root system to support the plants against the risk of stem breaking or lodging because of greater pressure from wind and sand. On the other hand, females need sturdier stem to provide stronger mechanical support for fruits and seeds during the breeding period, whereas males have relatively lower requirements for the mechanical support of stems because that inflorescences would drop off shortly after flowering (Yang et al., 2015). These indicated that the variation of basal diameter in males and females is inseparable from their growth environment and special reproductive strategies.

Morphological growth and biomass accumulation are regard as two important indices for evaluating plant growth and development, and the reduction of these traits usually means the cumulative effects of physiological function being damaged or inhibited (Xu et al., 2010; Chen et al., 2016). Therefore, we speculated that photosynthesis was severely compromised by wind erosion, because the physiological process was closely related to vegetative growth in plants. As expected, compared with the control condition, *P*_n_, *C_i_* and *T_r_* of all saplings decreased significantly in response to wind erosion (**Table 2**). The gas exchange reduction caused by wind erosion has been reported previously, such as *Calligonum mongolicum* (Fan et al., 2018), *Rhododendron ferrugineum* (Caldwell, 1970), and *Averrhoa carambola* (Marler and Zozor, 1992). We found that females always exhibited greater *P*_n_ than did males under either control or wind erosion. Moreover, the negative impact of wind erosion on males (58.52%) was greater than that on females (42.25%) in *P*_n_, which is in agreement with previous studies that *P*_n_ decreased more in weaker sex under the same stress intensity (Xu et al., 2008; He et al., 2016; Zhang et al., 2019). Chlorophyll fluorescence, as an indicator of photochemical efficiency of PSII, can provide insights into the degree of damage to photosynthetic apparatus under environmental stress (Maxwell and Johnson, 2000; Rohacek, 2002). In our study, under wind erosion condition, the lower *F*_v_/*F*_m_ and *Φ*_PSII_ observed in males suggested that males suffer more disorder in the electron transport chain of PSII. Thus, wind erosion inhibits photosynthetic capacity more in males than in females of *S. gordejevii*, as previously report in *S. paraplesia* during drought (Liao et al., 2019) and nocturnal warming (Liao et al., 2020).

### Females showed a better protective mechanism than males under wind erosion condition

Wind erosion is complex processes that alter the surrounding environment of plant growth, such as soil temperature, humidity, nutrient status and so on (Li et al., 2010; Liu et al., 2014a). Previous studies have confirmed that under environmental stress, such as high temperature, drought and high salinity, etc., RWC, MDA content, antioxidant enzymes system and osmotic regulators as a performance and protection mechanism would be changed to protect the normal growth of plants (Silva et al., 2010; Parida and Jha, 2013; Hatzig et al., 2014). In our study, we observed that wind erosion significantly decreased RWC of leaves but increased proline and soluble sugar content in all saplings, with females having significantly higher RWC but lower proline and soluble sugar content (**Figure 3A**, **Figure 4**). These results demonstrated wind erosion caused water shortage in plants, because wind erosion damaged the roots and exposed them to the air, thereby severely affected the function of water absorption (Yu et al., 2008; Luo and Zhao, 2015). As major constituents of osmoregulation, the increase of proline and soluble sugar content could effectively reduce cell osmotic pressure and prevent water loss, but also show more severe water shortage in males than that of in females (Zhang et al., 2012; Parida and Jha, 2013). Moreover, when plants exposed to adverse environmental constraints, excessive accumulation of reactive oxygen species (ROS) would eventually lead to oxidative stress, which is one of the important reasons of membrane lipid peroxidation under environmental stress (Jiang et al., 2013; He et al., 2016). The antioxidant enzyme system could effectively alleviate oxidative stress by regulating enzyme activity (such as SOD and POD), and higher enzyme activity also means that the plant has higher detoxicate and decompose capacity, as well as can suffer more oxidative damage caused by ROS (Gill and Tuteja, 2010; Qin et al., 2018). We found that wind erosion caused severe cellular membrane damage and oxidative stress, with MDA content, SOD and POD activity were significantly increased (**Figure 3B**, **Figure 5**). Meanwhile, females possessed higher antioxidant enzyme activity (SOD and POD activity) and lower MDA content, which suggesting there is a more effective detoxification and protective ability in females than in males under wind erosion condition. Sexually different responses in antioxidants under severe environmental stress were confirmed by those studies of *P. cathayana*, *M. alba* and *S. paraplesia* (Zhang et al., 2012; Qin et al., 2018; Liao et al., 2019). Additionally, the decrease of *P*_n_ and *F*_v_/*F*_m_ in our study could be attributed to serious water shortage and lipid peroxidation, since water and membrane could maintain the stable metabolism and cell internal environment (Mittler, 2002; Apel and Hirt, 2004; Qin et al., 2018).

On the other hand, biomass allocation is generally considered as the basis and vital parameter for understanding how adapt to environmental changes in plant life history (Li et al., 2010; Burylo et al., 2012; Tang et al., 2016). We observed that wind erosion altered the relative allocation of root, stem and leaf growth in both sexes, and more biomass was allocated to roots in females but allocated to leaves in males by reducing the biomass allocation of stems (**Figure 2**). Wind erosion, in general, means high light intensity, low moisture and nutrients. Females increased biomass allocation to roots to improve the ability of nutrient uptake and water and carbohydrates transportation in erosion condition, while males increased biomass allocation to leaf to utilize the highly available light resources and accumulate more photosynthetic products. Their different biomass accumulation strategies to cope with environmental changes might be attributable to males and females have different ecological evolutionary advantages that females usually show stronger resource absorption capacity, and males have higher resource utilization efficiency (Liu et al., 2021). Furthermore, as the most direct damage to plants caused by wind erosion is root bareness, distortion and splitting (Yu et al., 2008; Liu et al., 2014a; Liu et al., 2014b), females allocated more resources to roots could effectively resist the damage and better absorb moisture and mineral elements than males, which in line with the observations of Jiang et al. (2016) and Liao et al. (2020). Although more resources allocated to leaves in males compared to the control condition, there was no significant sexual difference of leaf percentage in total dry mass under the wind erosion condition. Hence, the biomass allocation pattern of females was more beneficial to resist wind erosion than that of males.

### Sexual differences in growth and physiology in response to wind erosion

Sexual differences in morphological growth, biomass accumulation and allocation, gas exchange, chlorophyll fluorescence, antioxidant enzyme activity, as well as osmotic regulation capacity under ambient or stressful environment have been widely reported in many dioecious plants (Chen et al., 2010a; Chen et al., 2010b; Juvany and Munne-Bosch, 2015; Randriamanana et al., 2015; Xu et al., 2015; Chen et al., 2016; Lei et al., 2017a; Retuerto et al., 2018; Ruuhola et al., 2018; Wu et al., 2018; Xia et al., 2020). We extended this conclusion to *S. gordejevii* by showing that males and females differed in their responses to wind erosion, with females having more resistance and effective mechanisms than males, which is in agreement with previous observations in *Salix* under environmental stress (Randriamanana et al., 2015; Jiang et al., 2016; Ruuhola et al., 2018; Liao et al., 2019; Liao et al., 2020). However, the results are contrary to the traditional theory that environmental stresses have a greater negative impact on females with higher reproductive costs, especially in contrast to *Populus* plants (Xu et al., 2008; Chen et al., 2010a; Chen et al., 2010b; Jiang et al., 2013; Xu et al., 2015; Lei et al., 2017a; Zhang et al., 2019; Liao et al., 2020). *Salix* and *populus*, belonging to Salicaceae, display an opposite resistance to environmental stress in both sexes. According to the results of previous studies, although *Salix* and *Populus* are the two closest genus of Salicaceae, there is no evidence for chromosomal rearrangements between chromosome XV (*Salix*) and XIX (*Populus*) of their sex determining genes, indicating that the sex determination loci in the willow and the poplar most likely do not share a common origin and has thus evolved separately (Pucholt et al., 2015). Moreover, Lei et al. (2017b) indicated that reproductive investments were dependent not only on sex, but also on species and environment. *P. purdomii* females at mid- and high-altitude have higher the relative reproductive investment, as well as *S. magnifica* females at mid-altitude. While no difference was observed for the relative reproductive investment in male and female *S. magnifica* at high-altitude. These showed that *Salix* and *Populus* have different reproductive investment strategies, and the willow would adjust their reproductive investment strategies according to environmental changes. Meanwhile, various compensatory mechanisms were found in *Salix* females to offset resource demands for production of fruits and seeds, including a high photosynthetic rate and an increased allocation to photosynthetic organs, which not only meet the resource needs of reproduction, but also enhance the resistance to environmental stress (Tozawa et al., 2009; Jiang et al., 2016; Lei et al., 2017b). In our study, we also observed that *S. gordejevii* females had higher LDM and *P*_n_ than did males under wind erosion conditions. Collectively, these evidences explain the reason that *S. gordejevii* females exhibit more resistance against wind erosion than do males. Furthermore, Yan et al. (2007) reported that the scattered *S. gordejevii* seeds germinated as soon as they contacted a moist surface, and wind erosion brought the ground surface closer to the groundwater. Thus, females had better growth and resistance to wind erosion than males in order to produce seeds better and germinate quickly through wind erosion after seed dispersal. Females bear the responsibility of breeding offspring, and their stable adaptability is conducive to the reproduction and development of the population.

## Conclusion

Wind erosion significantly reduced plant growth and photosynthesis, and seriously disrupted biomass allocation, osmotic regulation function and antioxidant enzyme system in *S. gordejevii* saplings, and led to significant sexual differences in these traits between males and females. Compared with males, females had higher growth and photosynthetic capacity, better water retention function and more efficient antioxidant system. Therefore, females look to be superior candidates for vegetation restoration, especially in shifting and semi-fixed dunes, because of their better growth and resistance against wind erosion under aeolian environment.

## Data availability statement

The original contributions presented in the study are included in the article/supplementary material. Further inquiries can be directed to the corresponding authors.

## Author contributions

GHL, SM, and XX conceived and designed the experiment. SM, LW, and GZL performed the experiment. SM analyzed the data. SM and XX wrote the paper. All authors contributed to the article and approved the submitted version.

## Funding

This work was supported by the Natural Science Foundation of Inner Mongolia Autonomous Region (2022LHQN03007), the Science and Technology Research Project of Colleges and Universities in Inner Mongolia Autonomous Region (NJZZ22589), and the Scientific Research Funding Project for Introduced High-level Talents of IMNU (2019YJRC018).

## Conflict of interest

The authors declare that the research was conducted in the absence of any commercial or financial relationships that could be construed as a potential conflict of interest.

## Publisher’s note

All claims expressed in this article are solely those of the authors and do not necessarily represent those of their affiliated organizations, or those of the publisher, the editors and the reviewers. Any product that may be evaluated in this article, or claim that may be made by its manufacturer, is not guaranteed or endorsed by the publisher.

## References

[B1] Anten N. P. Casado-Garcia R. Nagashima H. (2005). Effects of mechanical stress and plant density on mechanical characteristics, growth, and lifetime reproduction of tobacco plants. Am. Nat. 166 (6), 650−660. doi: 10.1086/497442 16475082

[B2] Apel K. Hirt H. (2004). Reactive oxygen species: metabolism, oxidative stress, and signal transduction. Annu. Rev. Plant Biol. 55, 373−399. doi: 10.1146/annurev.arplant.55.031903.141701 15377225

[B3] Bates L. S. Waldren R. P. Teare I. D. (1973). Rapid determination of free proline for water-stress studies. Plant Soil 39, 205−207. doi: 10.1007/bf00018060

[B4] Beauchamp C. Fridovich I. (1971). Superoxide dismutase: improved assays and an assay applicable to acrylamide gels. Anal. Biochem. 44, 276−287. doi: 10.1016/0003-2697(71)90370-8 4943714

[B5] Brugnoli E. Bjorkman O. (1992). Chloroplast movements in leaves: Influence on chlorophyll fluorescence and measurements of light-induced absorbance changes related to △pH and zeaxanthin formation. Photosynth. Res. 32 (1), 23−35. doi: 10.1007/BF00028795 24408152

[B6] Burylo M. Rey F. Dutoit T. (2012). Responses of five woody species to burial by marly sediment: the role of biomass allocation pattern flexibility. J. Plant Ecol. 5 (3), 287−293. doi: 10.1093/jpe/rtr030

[B7] Caldwell M. M. (1970). Plant gas exchange at high wind speeds. Plant Physiol. 46 (4), 535−537. doi: 10.1104/pp.46.4.535 16657501 PMC396631

[B8] Chen F. Chen L. Zhao H. Korpelainen H. Li C. (2010a). Sex-specific responses and tolerances of *Populus cathayana* to salinity. Physiol. Plantarum 140 (2), 163−173. doi: 10.1111/j.1399-3054.2010.01393.x 20561244

[B9] Chen M. Huang Y. Liu G. Qin F. Yang S. Xu X. (2016). Effects of enhanced UV-B radiation on morphology, physiology, biomass, leaf anatomy and ultrastructure in male and female mulberry (*Morus alba*) saplings. Environ. Exp. Bot. 129, 85−93. doi: 10.1016/j.envexpbot.2016.03.006

[B10] Chen L. Zhang S. Zhao H. Korpelainen H. Li C. (2010b). Sex-related adaptive responses to interaction of drought and salinity in *Populus yunnanensis* . Plant Cell Environ. 33 (10), 1767−1778. doi: 10.1111/j.1365-3040.2010.02182.x 20545878

[B11] Dech J. P. Maun M. A. (2006). Adventitious root production and plastic resource allocation to biomass determine burial tolerance in woody plants from central Canadian coastal dunes. Ann. Bot. 98 (5), 1095−1105. doi: 10.1093/aob/mcl196 17018567 PMC3292249

[B12] Ebell L. F. (1969). Variation in total soluble sugars of conifer tissues with method of analysis. Phytochemistry 8, 227−233. doi: 10.1016/s0031-9422(00)85818-5

[B13] Fan B. Zhao C. Zhang X. Sun K. (2018). Impacts of sand burial and wind erosion on regeneration and growth of a desert clonal shrub. Front. Plant Sci. 9, 1696. doi: 10.3389/fpls.2018.01696 30619381 PMC6297362

[B14] Gilbert M. E. Ripley B. S. (2010). Resolving the differences in plant burial responses. Austral Ecol. 35 (1), 53−59. doi: 10.1111/j.1442-9993.2009.02011.x

[B15] Gill S. S. Tuteja N. (2010). Reactive oxygen species and antioxidant machinery in abiotic stress tolerance in crop plants. Plant Physiol. Biochem. 48 (12), 909−930. doi: 10.1016/j.plaphy.2010.08.016 20870416

[B16] Hatzig S. Zaharia L. I. Abrams S. Hohmann M. Legoahec L. Bouchereau A.. (2014). Early osmotic adjustment responses in drought-resistant and drought-sensitive oilseed rape. J. Integr. Plant Biol. 56 (8), 797−809. doi: 10.1111/jipb.12199 24667002

[B17] He M. Shi D. Wei X. Hu Y. Wang T. Xie Y. (2016). Gender-related differences in adaptability to drought stress in the dioecious tree *Ginkgo biloba* . Acta Physiol. Plant 38, 124. doi: 10.1007/s11738-016-2148-0

[B18] Hodges D. M. DeLong J. M. Forney C. F. Prange R. K. (1999). Improving the thiobarbituric acid-reactive-substances assay for estimating lipid peroxidation in plant tissues containing anthocyanin and other interfering compounds. Planta 207 (4), 604−611. doi: 10.1007/s004250050524 28456836

[B19] Jiang H. Korpelainen H. Li C. (2013). *Populus yunnanensis* males adopt more efficient protective strategies than females to cope with excess zinc and acid rain. Chemosphere 91 (8), 1213−1220. doi: 10.1016/j.chemosphere.2013.01.041 23415309

[B20] Jiang H. Zhang S. Lei Y. Xu G. Zhang D. (2016). Alternative growth and defensive strategies reveal potential and gender specific trade-offs in dioecious plants *Salix paraplesia* to nutrient availability. Front. Plant Sci. 7, 1064. doi: 10.3389/fpls.2016.01064 27489556 PMC4951494

[B21] Juvany M. Munne-Bosch S. (2015). Sex-related differences in stress tolerance in dioecious plants: a critical appraisal in a physiological context. J. Exp. Bot. 66 (20), 6083−6092. doi: 10.1093/jxb/erv343 26163697

[B22] Kubo S. Hashida K. Makino R. Magara K. Kenzo T. Kato A.. (2013). Chemical composition of desert willow (*Salix psammophila*) grown in the Kubuqi Desert, Inner Mongolia, China: bark extracts associated with environmental adaptability. J. Agric. Food Chem. 61 (50), 12226−12231. doi: 10.1021/jf4038634 24274758

[B23] Lei Y. Chen K. Jiang H. Yu L. Duan B. (2017a). Contrasting responses in the growth and energy utilization properties of sympatric *Populus* and *Salix* to different altitudes: implications for sexual dimorphism in Salicaceae. Physiol. Plantarum 159 (1), 30−41. doi: 10.1111/ppl.12479 27300648

[B24] Lei Y. Jiang Y. Chen K. Duan B. Zhang S. Korpelainen H.. (2017b). Reproductive investments driven by sex and altitude in sympatric *Populus* and *Salix* trees. Tree Physiol. 37 (11), 1503−1514. doi: 10.1093/treephys/tpx075 28985430

[B25] Levin N. Kidron G. J. Ben-Dor E. (2008). A field quantification of coastal dune perennial plants as indicators of surface stability, erosion or deposition. Sedimentology 55 (4), 751−772. doi: 10.1111/j.1365-3091.2007.00920.x

[B26] Liao J. Cai Z. Song H. Zhang S. (2020). Poplar males and willow females exhibit superior adaptation to nocturnal warming than the opposite sex. Sci. Total Environ. 717, 137179. doi: 10.1016/j.scitotenv.2020.137179 32062275

[B27] Liao J. Song H. Tang D. Zhang S. (2019). Sexually differential tolerance to water deficiency of *Salix paraplesia*-a female-biased alpine willow. Ecol. Evol. 9 (15), 8450−8464. doi: 10.1002/ece3.5175 31410253 PMC6686310

[B28] Liu M. Z. Jiang G. M. Li Y. G. Gao L. M. Niu S. L. Cui H. X.. (2003). Gas exchange, photochemical efficiency, and leaf water potential in three *Salix* species. Photosynthetica 41 (3), 393−398. doi: 10.1023/b:phot.0000015463.04706.f3

[B29] Liu M. Korpelainen H. Li C. (2021). Sexual differences and sex ratios of dioecious plants under stressful environments. J. Plant Ecol. 14 (5), 920−933. doi: 10.1093/jpe/rtab038

[B30] Liu B. Liu Z. Lü X. Maestre F. T. Wang L. (2014a). Sand burial compensates for the negative effects of erosion on the dune-building shrub *Artemisia wudanica* . Plant Soil 374, 263−273. doi: 10.1007/s11104-013-1866-y

[B31] Liu B. Liu Z. Wang L. Wang Z. (2014b). Responses of rhizomatous grass *Phragmites communis* to wind erosion: effects on biomass allocation. Plant Soil 380, 389−398. doi: 10.1007/s11104-014-2104-y

[B32] Li S. Werger M. J. A. Zuidema P. A. Yu F. Dong M. (2010). Seedlings of the semi-shrub *Artemisia ordosica* are resistant to moderate wind denudation and sand burial in Mu Us sandland, China. Trees-Struct. Funct. 24 (3), 515−521. doi: 10.1007/s00468-010-0422-0

[B33] Luo W. Zhao W. (2015). Effects of wind erosion and sand burial on growth and reproduction of a clonal shrub. Flora 217, 164−169. doi: 10.1016/j.flora.2015.10.006

[B34] Luo W. Zhao W. Zhuang Y. (2018). Sand-burial and wind erosion promote oriented-growth and patchy distribution of a clonal shrub in dune ecosystems. Catena 167, 212−220. doi: 10.1016/j.catena.2018.04.043

[B35] Maehly A. C. Chance B. (1954). The assay of catalases and peroxidases. Methods Biochem. Anal. 1, 357−424. doi: 10.1002/9780470110171.ch14 13193536

[B36] Marler T. E. Zozor Y. (1992). Carambola growth and leaf gas-exchange responses to seismic or wind stress. Hortscience 27 (8), 913−915. doi: 10.21273/Hortsci.27.8.913

[B37] Maxwell K. Johnson G. N. (2000). Chlorophyll fluorescence–a practical guide. J. Exp. Bot. 51 (345), 659−668. doi: 10.1093/jxb/51.345.659 10938857

[B38] Melnikova N. V. Borkhert E. V. Snezhkina A. V. Kudryavtseva A. V. Dmitriev A. A. (2017). Sex-specific response to stress in *Populus* . Front. Plant Sci. 8, 1827. doi: 10.3389/fpls.2017.01827 29123538 PMC5662629

[B39] Mittler R. (2002). Oxidative stress, antioxidants and stress tolerance. Trends Plant Sci. 7 (9), 405−410. doi: 10.1016/s1360-1385(02)02312-9 12234732

[B40] Obeso J. R. (2002). The costs of reproduction in plants. New Phytol. 155 (3), 321−348. doi: 10.1046/j.1469-8137.2002.00477.x 33873312

[B41] Parida A. K. Jha B. (2013). Physiological and biochemical responses reveal the drought tolerance efficacy of the halophyte *Salicornia brachiata* . J. Plant Growth Regul. 32, 342−352. doi: 10.1007/s00344-012-9303-7

[B42] Perumal V. J. Maun M. A. (2006). Ecophysiological response of dune species to experimental burial under field and controlled conditions. Plant Ecol. 184 (1), 89−104. doi: 10.1007/s11258-005-9054-7

[B43] Pucholt P. Ronnberg-Wastljung A. C. Berlin S. (2015). Single locus sex determination and female heterogamety in the basket willow (*Salix viminalis* L.). Heredity 114, 575−583. doi: 10.1038/hdy.2014.125 25649501 PMC4434249

[B44] Qin F. Liu G. Huang G. Dong T. Liao Y. Xu X. (2018). Zinc application alleviates the adverse effects of lead stress more in female *Morus alba* than in males. Environ. Exp. Bot. 146, 68−76. doi: 10.1016/j.envexpbot.2017.10.003

[B45] Randriamanana T. R. Nissinen K. Moilanen J. Nybakken L. Julkunen-Tiitio R. (2015). Long-term UV-B and temperature enhancements suggest that females of *Salix myrsinifolia* plants are more tolerant to UV-B than males. Environ. Exp. Bot. 109, 296−305. doi: 10.1016/j.envexpbot.2014.06.007

[B46] Renner S. S. Ricklefs R. E. (1995). Dioecy and its correlates in the flowering plants. Am. J. Bot. 82 (5), 596−606. doi: 10.2307/2445418

[B47] Retuerto R. Vilas J. S. Varga S. (2018). Sexual dimorphism in response to stress. Environ. Exp. Bot. 146, 1−4. doi: 10.1016/j.envexpbot.2017.12.006

[B48] Rohacek K. (2002). Chlorophyll fluorescence parameters: the definitions, photosynthetic meaning, and mutual relationships. Photosynthetica 40 (1), 13−29. doi: 10.1023/A:1020125719386

[B49] Ruuhola T. Nybakken L. Randriamanana T. Lavola A. Julkunen-Tiitto R. (2018). Effects of long-term UV-exposure and plant sex on the leaf phenoloxidase activities and phenolic concentrations of *Salix myrsinifolia* (Salisb.). Plant Physiol. Biochem. 126, 55−62. doi: 10.1016/j.plaphy.2018.02.025 29501893

[B50] Shi P. Yan P. Yuan Y. Nearing M. A. (2004). Wind erosion research in China: past, present and future. Prog. Phys. Geogr. 28 (3), 366−386. doi: 10.1191/0309133304pp416ra

[B51] Silva E. N. Ferreira-Silva S. L. Fontenele Ade V. Ribeiro R. V. Viegas R. A. Silveira J. A. (2010). Photosynthetic changes and protective mechanisms against oxidative damage subjected to isolated and combined drought and heat stresses in *Jatropha curcas* plants. J. Plant Physiol. 167 (14), 1157−1164. doi: 10.1016/j.jplph.2010.03.005 20417989

[B52] Su H. Li Y. Lan Z. Xu H. Liu W. Wang B.. (2009). Leaf-level plasticity of *Salix gordejevii* in fixed dunes compared with lowlands in Hunshandake Sandland, North China. J. Plant Res. 122, 611−622. doi: 10.1007/s10265-009-0249-1 19536609

[B53] Tang J. Busso C. A. Jiang D. Musa A. Wu D. Wang Y.. (2016). Experimental sand burial affects seedling survivorship, morphological traits, and biomass allocation of *Ulmus pumila* var. *sabulosa* in the Horqin Sandy Land, China. Solid Earth 7 (4), 1085−1094. doi: 10.5194/se-7-1085-2016

[B54] Tozawa M. Ueno N. Seiwa K. (2009). Compensatory mechanisms for reproductive costs in the dioecious tree *Salix integra* . Botany 87 (3), 315−323. doi: 10.1139/B08-125

[B55] Ward J. K. Dawson T. E. Ehleringer J. R. (2002). Responses of *Acer negundo* genders to interannual differences in water availability determined from carbon isotope ratios of tree ring cellulose. Tree Physiol. 22 (5), 339–346. doi: 10.1093/treephys/22.5.339 11960758

[B56] Weatherley P. E. (1950). Studies in the water relations of the cotton plant I. the field measurement of water deficits in leaves. New Phytol. 49, 81–97. doi: 10.1111/j.1469-8137.1950.tb05146.x

[B57] Wu Q. Tang Y. Dong T. Liao Y. Li D. He X.. (2018). Additional AM fungi inoculation increase *Populus cathayana* intersexual competition. Front. Plant Sci. 9, 607. doi: 10.3389/fpls.2018.00607 29868065 PMC5951968

[B58] Xia Z. He Y. Yu L. Lv R. Korpelainen H. Li C. (2020). Sex-specific strategies of phosphorus (P) acquisition in *Populus cathayana* as affected by soil P availability and distribution. New Phytol. 225 (2), 782−792. doi: 10.1111/nph.16170 31487045

[B59] Xu X. Li Y. Wang B. Hu J. Liao Y. (2015). Salt stress induced sex-related spatial heterogeneity of gas exchange rates over the leaf surface in *Populus cathayana* Rehd. Acta Physiol. Plant 37, 1079. doi: 10.1007/s11738-014-1709-3

[B60] Xu X. Peng G. Wu C. Korpelainen H. Li C. (2008). Drought inhibits photosynthetic capacity more in females than in males of *Populus cathayana* . Tree Physiol. 28 (11), 1751−1759. doi: 10.1093/treephys/28.11.1751 18765380

[B61] Xu X. Zhao H. Zhang X. Hanninen H. Korpelainen H. Li C. (2010). Different growth sensitivity to enhanced UV-B radiation between male and female *Populus cathayana* . Tree Physiol. 30 (12), 1489−1498. doi: 10.1093/treephys/tpq094 21071771

[B62] Yang Y. He X. Xu X. Yang D. (2015). Scaling relationships among twig components are affected by sex in the dioecious tree *Populus cathayana* . Trees-Struct. Funct. 29, 737−746. doi: 10.1007/s00468-014-1151-6

[B63] Yan Q. Liu Z. Ma J. Jiang D. (2007). The role of reproductive phenology, seedling emergence and establishment of perennial *Salix gordejevii* in active sand dune fields. Ann. Bot. 99 (1), 19−28. doi: 10.1093/aob/mcl228 17085475 PMC2802971

[B64] Yu F. Wang N. He W. Chu Y. Dong M. (2008). Adaptation of rhizome connections in drylands: increasing tolerance of clones to wind erosion. Ann. Bot. 102 (4), 571−577. doi: 10.1093/aob/mcn119 18621966 PMC2701773

[B65] Zhang S. Chen L. Duan B. Korpelainen H. Li C. (2012). *Populus cathayana* males exhibit more efficient protective mechanisms than females under drought stress. For. Eco. Manage. 275, 68−78. doi: 10.1016/j.foreco.2012.03.014

[B66] Zhang R. Liu J. Liu Q. He H. Xu X. Dong T. (2019). Sexual differences in growth and defence of *Populus yunnanensis* under drought stress. Can. J. For. Res. 49 (5), 491−499. doi: 10.1139/cjfr-2018-0270

